# Supplementary material for: Quality improvement bundles to decrease hypothermia in very low/extremely low birth weight infants at birth: a systematic review and meta-analysis
Source: PeerJ. 2024 Nov 1;12:e18425. doi: 10.7717/peerj.18425 (PMC11533904; doi:10.7717/peerj.18425)
Supplement: Supplemental Information 2 [file peerj-12-18425-s002.docx]

**Search Strategy**

**Pubmed**

#1 ((("Infant, Very Low Birth Weight"[Mesh]) OR "Infant, Extremely Low Birth Weight"[Mesh]) AND ("Hypothermia"[Mesh])) AND ("Quality Improvement"[Mesh])

#2 (((((((very-low-birth-weight Infant*[Title/Abstract]) OR (Infant*, very-low-birth-weight[Title/Abstract])) OR (very low birth weight Infant*[Title/Abstract])) OR (Infant*, very low birth weight[Title/Abstract])) OR (very low birth weight[Title/Abstract])) OR (very-low-birth-weight[Title/Abstract])) OR ((((((extremely-low-birth-weight Infant*[Title/Abstract]) OR (Infant*, extremely-low-birth-weight[Title/Abstract])) OR (extremely low birth weight Infant*[Title/Abstract])) OR (Infant*, extremely low birth weight[Title/Abstract])) OR (extremely low birth weight[Title/Abstract])) OR (extremely-low-birth-weight[Title/Abstract]))) AND (((((((hypothermia*[Title/Abstract]) OR (hypothermia*, accidental[Title/Abstract])) OR (accidental hypothermia*[Title/Abstract])) OR (admission hypothermia*[Title/Abstract])) OR (admission temperature[Title/Abstract])) OR (normothermia[Title/Abstract])) OR (thermoregulation*[Title/Abstract]))

#3 #1 OR #2

**Cochrane Library**

#1 MeSH descriptor: [Infant, Very Low Birth Weight] explode all trees

#2 MeSH descriptor: [Infant, Extremely Low Birth Weight] explode all trees

#3 #1 OR #2

#4 MeSH descriptor: [Hypothermia] explode all trees

#5 #3 AND #4

#6 ((very-low-birth-weight Infant*) OR (Infant*, very-low-birth-weight) OR (very low birth weight Infant*) OR (Infant*, very low birth weight) OR (very low birth weight) OR (very-low-birth-weight)):ti,ab,kw (Word variations have been searched)

#7 ((extremely-low-birth-weight Infant*) OR (Infant*, extremely-low-birth-weight) OR (extremely low birth weight Infant*) OR (Infant*, extremely low birth weight) OR (extremely low birth weight) OR (extremely-low-birth-weight)):ti,ab,kw (Word variations have been searched)

#8 #6 OR #7

#9 ((hypothermia*) OR (hypothermia*, accidental) OR (accidental hypothermia*) OR (admission hypothermia*) OR (admission temperature) OR (normothermia) OR (thermoregulation*)):ti,ab,kw (Word variations have been searched)

#10 #8 AND #9

**Embase**

#1 'very low birth weight'/exp

#2 'very-low-birth-weight infant*':ab,ti OR 'infant*, very-low-birth-weight':ab,ti OR 'very low birth weight infant*':ab,ti OR 'infant*, very low birth weight':ab,ti OR 'very low birth weight':ab,ti

#3 'extremely low birth weight'/exp

#4 'extremely-low-birth-weight infant*':ab,ti OR 'infant*, extremely-low-birth-weight':ab,ti OR 'extremely low birth weight infant*':ab,ti OR 'infant*, extremely low birth weight':ab,ti OR 'extremely low birth weight':ab,ti

#5 #1 OR #2 OR #3 OR #4

#6 'hypothermia'/exp

#7 hypothermia*:ab,ti OR 'hypothermia*, accidental':ab,ti OR 'accidental hypothermia*':ab,ti OR 'admission hypothermia*':ab,ti OR 'admission temperature':ab,ti OR normothermia:ab,ti OR thermoregulation*:ab,ti

#8 #6 OR #7

#9 #5 AND #8

**Web of Science**

#1 TS=(very-low-birth-weight Infant* ) OR TS=(Infant*, very-low-birth-weight) OR TS=(very low birth weight Infant*) OR TS=(Infant*, very low birth weight ) OR TS=(very low birth weight) OR TS=(very-low-birth-weight)

#2 TS=(extremely-low-birth-weight Infant* ) OR TS=(Infant*, extremely-low-birth-weight ) OR TS=(extremely low birth weight Infant*) OR TS=(Infant*, extremely low birth weight ) OR TS=(extremely low birth weight) OR TS=(extremely-low-birth-weight)

#3 #1 OR #2

#4 TS=(hypothermia*) OR TS=(hypothermia*, accidental) OR TS=(accidental hypothermia*) OR TS=(admission hypothermia*) OR TS=(admission temperature) OR TS=(normothermia) OR TS=(thermoregulation*)

#5 #3 AND #4
