# Supplementary material for: Quality improvement bundles to decrease hypothermia in very low/extremely low birth weight infants at birth: a systematic review and meta-analysis
Source: PeerJ. 2024 Nov 1;12:e18425. doi: 10.7717/peerj.18425 (PMC11533904; doi:10.7717/peerj.18425)
Supplement: Supplemental Information 3 [file peerj-12-18425-s003.doc]

**The risk of bias in each included study.** The included studies were evaluated using QI-MQCS.

| Study | D1 | D2 | D3 | D4 | D5 | D6 | D7 | D8 | D9 | D10 | D11 | D12 | D13 | D14 | D15 | D16 | Total | Study Quality |
| --- | --- | --- | --- | --- | --- | --- | --- | --- | --- | --- | --- | --- | --- | --- | --- | --- | --- | --- |
| Kent 2008 | 1 | 1 | 1 | 0 | 1 | 1 | 1 | 1 | 1 | 0 | 1 | 0 | 0 | 0 | 0 | 1 | 10 | medium |
| Lee 2008 | 1 | 1 | 1 | 0 | 1 | 1 | 1 | 0 | 1 | 0 | 1 | 0 | 1 | 1 | 0 | 0 | 9 | medium |
| Billimoria 2013 | 1 | 1 | 1 | 1 | 1 | 1 | 1 | 0 | 1 | 0 | 1 | 1 | 1 | 0 | 0 | 1 | 12 | high |
| DeMauro 2013 | 1 | 1 | 1 | 1 | 1 | 1 | 1 | 0 | 1 | 0 | 1 | 0 | 1 | 0 | 0 | 1 | 11 | high |
| Godfrey 2013 | 1 | 1 | 1 | 1 | 1 | 1 | 1 | 0 | 1 | 0 | 1 | 0 | 1 | 0 | 0 | 1 | 10 | medium |
| Manani 2013 | 1 | 1 | 1 | 1 | 1 | 1 | 1 | 1 | 1 | 1 | 1 | 1 | 1 | 1 | 1 | 0 | 15 | high |
| Castrodale 2014 | 1 | 1 | 1 | 1 | 1 | 1 | 1 | 1 | 1 | 0 | 1 | 0 | 0 | 0 | 0 | 0 | 10 | medium |
| Pinheiro 2014 | 1 | 1 | 1 | 0 | 1 | 1 | 1 | 1 | 1 | 1 | 1 | 1 | 1 | 1 | 1 | 1 | 15 | high |
| Sivanaridan 2016 | 1 | 1 | 1 | 1 | 1 | 1 | 1 | 1 | 1 | 0 | 1 | 1 | 0 | 0 | 1 | 1 | 12 | high |
| Yip 2017 | 1 | 1 | 1 | 1 | 1 | 1 | 1 | 0 | 1 | 0 | 1 | 1 | 1 | 1 | 0 | 0 | 12 | high |
| Caldas 2018 | 1 | 1 | 1 | 1 | 1 | 1 | 1 | 1 | 1 | 0 | 1 | 0 | 0 | 0 | 0 | 1 | 11 | high |
| Bhatt 2020 | 1 | 1 | 1 | 1 | 1 | 1 | 1 | 0 | 1 | 0 | 1 | 0 | 1 | 0 | 0 | 1 | 11 | high |
| Croop 2020 | 1 | 1 | 1 | 1 | 1 | 1 | 1 | 0 | 1 | 0 | 1 | 0 | 0 | 0 | 1 | 1 | 11 | high |
| Dixon 2021 | 1 | 1 | 1 | 1 | 1 | 1 | 1 | 1 | 1 | 1 | 1 | 0 | 1 | 0 | 0 | 1 | 13 | high |
| Frazer 2021 | 1 | 1 | 1 | 1 | 1 | 0 | 1 | 1 | 1 | 0 | 1 | 0 | 0 | 0 | 0 | 1 | 10 | medium |
| Young 2021 | 1 | 1 | 1 | 1 | 1 | 1 | 1 | 1 | 1 | 0 | 1 | 1 | 0 | 1 | 1 | 1 | 14 | high |
| Bi 2022 | 1 | 1 | 1 | 1 | 1 | 1 | 1 | 1 | 1 | 1 | 1 | 1 | 1 | 0 | 0 | 1 | 14 | high |
| Singh 2022 | 1 | 1 | 1 | 1 | 1 | 1 | 1 | 1 | 1 | 0 | 1 | 0 | 0 | 1 | 1 | 1 | 13 | high |

QI-MQCS: minimum quality standards for quality improvement; D1: Organizational Motivation; D2: Intervention Rationale; D3: Intervention Description; D4: Organizational Characteristics; D5: Implementation; D6: Study Design; D7: Comparator Description; D8: Data Sources; D9: Timing; D10: Adherence/Fidelity; D11: Health Outcomes; D12: Organizational Readiness; D13: Penetration/Reach; D14: Sustainability; D15: Spread; D16: Limitations.

Three grades of quality were used for each study, based on the score achieved in the critical appraisals: > 10 was high quality, 7~10 was medium quality, and ﹤7 was low quality.
