# Supplementary figures and images for: Quality improvement bundles to decrease hypothermia in very low/extremely low birth weight infants at birth: a systematic review and meta-analysis
Source: PeerJ. 2024 Nov 1;12:e18425. doi: 10.7717/peerj.18425 (PMC11533904; doi:10.7717/peerj.18425)

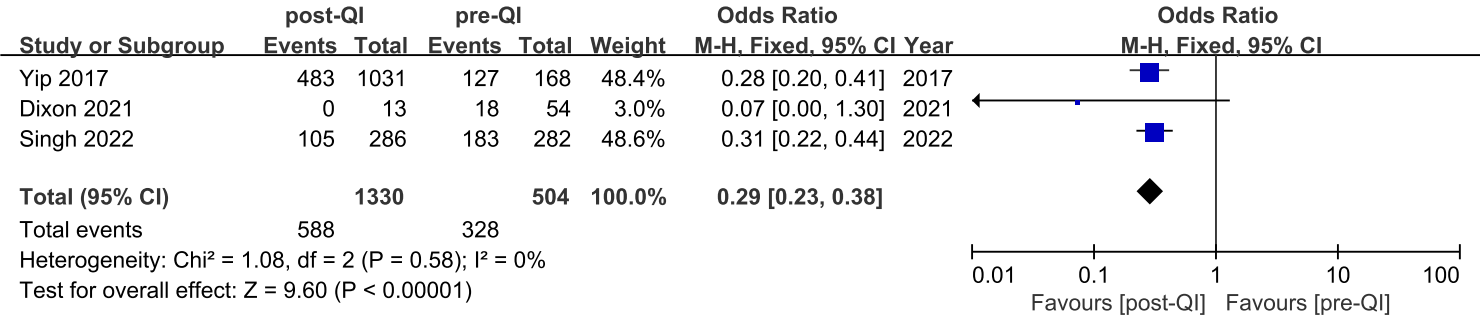

Supplement: Supplemental Information 4 [file peerj-12-18425-s004.pdf]

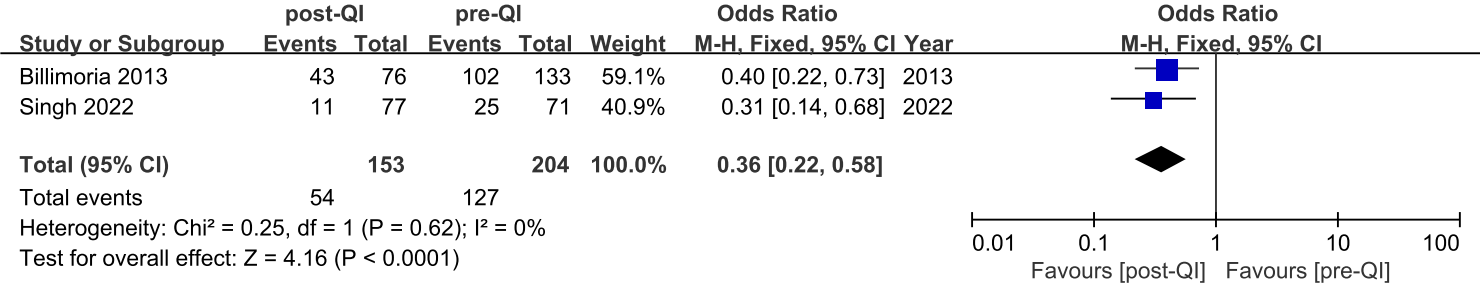

Supplement: Supplemental Information 5 [file peerj-12-18425-s005.pdf]
