# Supplementary material for: Quality improvement bundles to decrease hypothermia in very low/extremely low birth weight infants at birth: a systematic review and meta-analysis
Source: PeerJ. 2024 Nov 1;12:e18425. doi: 10.7717/peerj.18425 (PMC11533904; doi:10.7717/peerj.18425)
Supplement: Supplemental Information 8 [file peerj-12-18425-s008.docx]

**The rationale for conducting the systematic review / meta-analysis**

Hypothermia in preterm infants correlates with increased morbidity and mortality, especially among those with very low or extremely low birth weights (VLBW/ELBW). An increasing number of healthcare facilities are implementing quality improvement (QI) bundles to lower the incidence of hypothermia at birth in this vulnerable population. However, the effectiveness and safety of these interventions have yet to be fully assessed. A meta-analysis is necessary to evaluate the efficacy and safety of QI bundles in reducing hypothermia at birth among VLBW/ELBW infants.

**The contribution that it makes to knowledge in light of previously published related reports, including other meta-analyses and systematic reviews**

This meta-analysis is the first to quantitatively evaluate the efficacy and safety of QI bundles in reducing hypothermia among VLBW/ELBW infants at birth. We quantitatively assessed the incidence rates of mild hypothermia, moderate hypothermia, and hyperthermia pre- and post-QI.
